# Unilateral hemi-central retinal artery occlusion as a presenting sign of Susac syndrome

**DOI:** 10.5935/0004-2749.2022-0195

**Published:** 2024-07-16

**Authors:** Kenzo Hokazono, Marília da Cruz Fagundes, Alessandra Filpo Ferreira da Silva, Camila Feijó Minku, Bernardo Corrêa de Almeida Teixeira, Mário Luiz Ribeiro Monteiro

**Affiliations:** 1 Department of Ophthalmology, Faculdade de Medicina, Universidade de São Paulo, São Paulo, SP, Brazil; 2 Department of Radiology, Hospital de Clínicas, Universidade Federal do Paraná, Curitiba, PR, Brazil; 3 Department of Neurology, Hospital de Clínicas, Universidade Federal do Paraná, Curitiba, PR, Brazil

**Keywords:** Ophthalmological diagnostic techniques, Retinal artery occlusion, Vertigo, Cerebrovascular disorders, Susac syndrome

## Abstract

A young woman presented at our clinic with sudden visual loss in the right eye, recurrent vertigo, and right-sided tinnitus. We performed a complete ophthalmological evaluation. This revealed effects of the condition on the small arterioles of the peripheral retina. Susac syndrome is characterized by the clinical triad of retinal arteriolar occlusions, cochleovestibular manifestations, and encephalopathy (which can be identified by neuroimaging abnormalities). Early diagnosis and immunosuppressive therapy improved the patient's visual acuity and the remission of her other symptoms. Hemi-central retinal artery occlusion is an atypical neuro-ophthalmological finding in this disease. However, its identification as a sign of Susac syndrome may facilitate timely diagnosis and accurate treatment.

## INTRODUCTION

Susac syndrome (SS) is a rare autoimmune inflammatory endotheliopathy that causes microangiopathy and leads to vascular occlusions and ischemia in the arterioles of the retina, inner ear, and brain^(1)^.

The clinical triad of branch retinal arterial occlusions (BRAO), hearing loss, and brain involvement (encephalopathy, focal neurological deficits, or headache) is considered pathognomonic^(2)^. However, only 15% of patients present with the complete triad^(3)^.

For diagnosis, typical brain magnetic resonance imaging (MR1) findings are also required. These include fluid-attenuated inversion recovery (FLAIR) and T2 hyperintense, small, round multifocal snowball-like lesions of the white matter, at least one of which is in the corpus callosum. Leptomeningeal enhancement may also be observed.

## CASE REPORT

A 27-year-old woman presented with a sudden onset of visual loss in the right eye (OD). She also complained of recurrent episodes of vertigo, right-sided tinnitus, and auricular plenitude sensations over the previous week.

**Figure 1:**
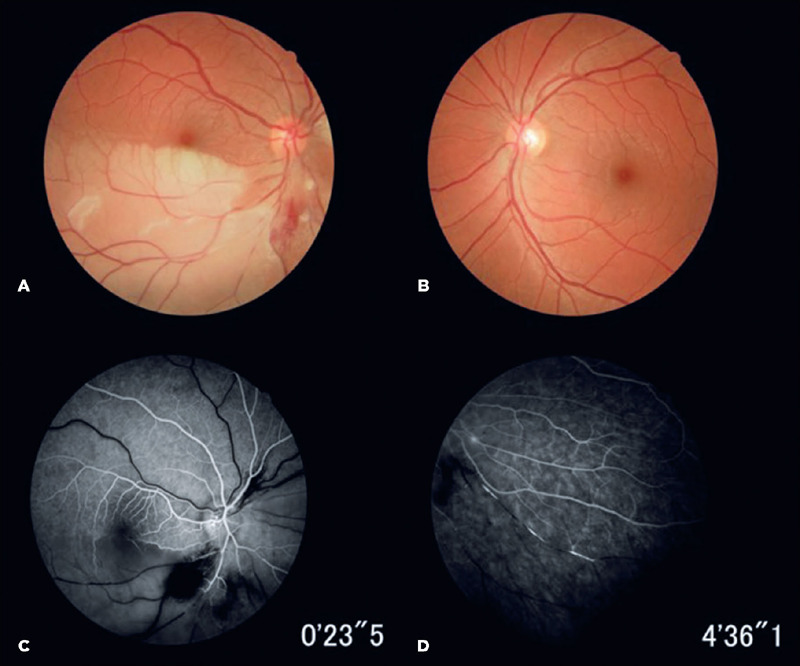
Color fundus photographs from a patient with Susac syndrome. The first image shows the extensive retinal edema found in the inferior half of the right eye, sparing the fovea (A). This was absent in the left eye (B). An occlusion of the inferior arterial hemi-trunk of the central retinal artery was apparent and fluorescein angiography showed a hemi-central retinal artery occlusion in the right eye (C) and multiple smaller branch retinal arterial occlusions on both sides (D), with non-filling of the retinal vessels and typical hyper-fluorescence of the arteriolar vessel walls in both eyes.

Ophthalmic examination revealed a best-corrected visual acuity of 20/20 in both eyes (OU). Fundus examination identified a hemi-central retinal artery occlusion (CRAO) in the OD. Fundus fluorescein angiography (FA) confirmed an ipsilateral occlusion of the inferior arterial hemi-trunk and hyper-fluorescence was observed in the arteriolar vessel walls, with multiple smaller BRAOs in the OU (Figure 1).

**Figure 2:**
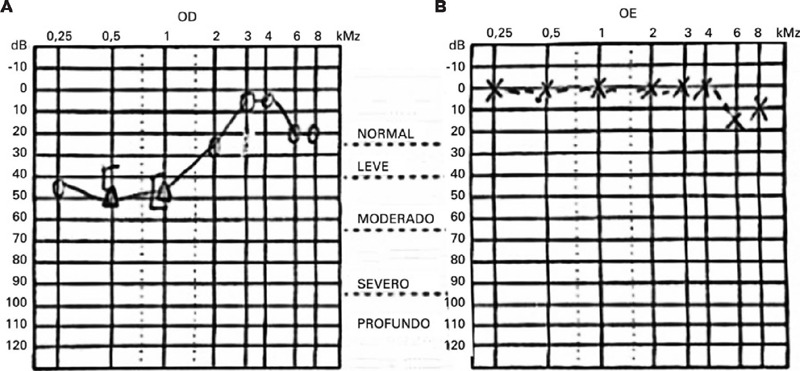
An audiogram of a patient with Susac syndrome. The reading showed typical low-tone (40 dB) mild sensorineural hearing loss in the right ear (A). The hearing thresholds of the left ear were within normal limits (B).

A Rinne test yielded positive results on both sides, and a Weber test lateralized to the left ear, suggesting sensorineural hearing loss. An audiogram confirmed low-frequency sensorineural hearing loss in the right ear (Figure 2).

**Figure 3:**
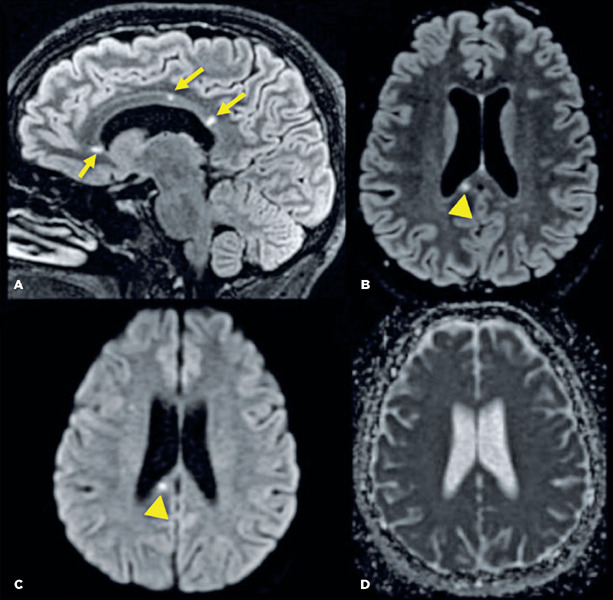
Brain magnetic resonance imaging of a patient with Susac syndrome. Multiple small round lesions with hyperintensity on fluid-attenuated inversion recovery (A, B) were seen in the white matter, particularly within the central fibers of the corpus callosum (arrows). There was no involvement of the ependymal undersurface. These characteristics are typical of the "snowball" lesions seen in Susac syndrome. The dominant lesion in the splenium (arrowhead) showed restricted diffusion on diffusion-weighted imaging (C) and an apparent diffusion coefficient map (D), suggesting a small ischemic infarct.

A brain MR1 of the patient revealed small spherical high-signal intensity lesions on FLAIR and T2 sequences. These were located within the fiber tracts of the corpus callosum, with no ependymal undersurface involvement. The dominant lesion in the splenium showed restricted diffusion indicative of a small ischemic infarct (Figure 3).

A workup was performed to exclude vascular and embolic diseases. This included complete blood count; erythrocyte sedimentation rate; levels of C-reactive protein, antinuclear antibodies, serum complement, 1gG and 1gM anticardiolipin antibodies, and lupus anticoagulant. All of these were within the normal range. The results of an analysis of the cerebrospinal fluid, a transthoracic echocardiogram, and a Doppler ultrasound of cervical arteries were all unremarkable.

The presence of the clinical triad of multiple retinal artery occlusion, cochleovestibular involvement, and brain abnormalities, confirmed by MR1 established the diagnosis as SS.

**Figure 4:**
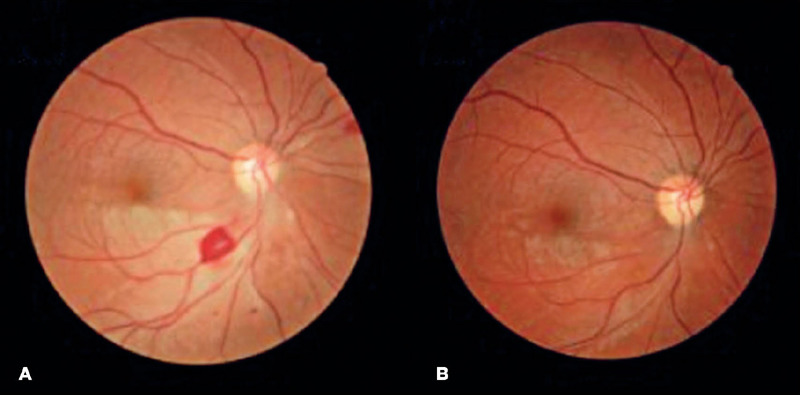
Color fundus photographs from a patient with Susac syndrome after immunosuppressive therapy. At the first follow-up, there was a reduction in the extensive retinal edema and opacity of the right eye and partial ipsilateral revascularization (A). The subsequent follow-up examination revealed neither retinal edema nor hemorrhage (B).

The patient was treated with endovenous high dose methylprednisolone and discharged with oral azathioprine and a prednisone tapering plan. At her two-month follow-up visit, she reported an improvement in her visual acuity and no additional symptoms. A fundus examination revealed a reduction in retinal edema and opacity in the OD and partial ipsilateral revascularization (Figure 4A), which was no longer apparent at the next follow-up (Figure 4B).

## DISCUSSION

In 1979, Susac et al.^(1)^ described a syndrome involving microangiopathy of the brain and retina. The authors reported two cases, both young women, who presented with psychosis, hearing loss, and multiple BRAO. Subsequently, Coppeto et al.^(4)^ and Monteiro et al.^(2)^ reported four more cases, allowing a comprehensive characterization of the syndrome as a distinct entity. In these reports, the authors highlighted BRAO as a clinical feature of SS, which was present in both eyes in five of the six reported cases. Since then, other case reports and case series have been published, emphasizing BRAO as a key clinical feature of SS.

Nowadays, SS is classically associated with impairment of the peripheral retina, causing multiple recurrent BRAO and yellow, non-refractile or refractile, retinal arterial wall plaques (Gass plaques). These are found in mid-arteriolar segments visible as fluorescence on fundus FA^(5)^. 1t is likely that the endothelial injury in SS causes turbulent flow, leading to fibrin-like white material deposits in the vessel wall, (which is different from platelet emboli, which accumulate at the bifurcation)^(2)^, predisposing it to *in situ* thrombosis^(6)^. Therefore, to detect this peripheral retinal microangiopathy, a comprehensive fundus examination of both eyes, including FA, should be performed. Attention should be paid to any damage to small arterioles. This should be accompanied by further otological evaluation and careful neuroimaging analysis.

As SS usually presents with a preference for peripheral retinal arterioles, only a few cases have been reported in which the central retinal artery or its first branch was initially occluded^(2)^. Hemi-CRAO is very unusual in SS and was the presenting sign in our case, making it unique.

Adatia et al.^(7)^ have described the case of a 36-year-old woman who presented with CRAO one month after the onset of neurological symptoms, including episodic blurred speech, decreased hearing, paresthesia, and weakness. Apostolos-Pereira et al.^(5)^ have reported a case of a 24-year-old man with CRAO in the left eye as the sole presenting sign of SS. Three weeks later, the patient developed hearing loss, encephalopathy and multiple BRAO in the right eye.

Buelens et al.^(8)^ recently published the case of a 22-year-old woman with CRAO in one eye and a negative comprehensive workup. After ruling out all neurological diseases and treating the patient empirically, an audiogram was performed, revealing mild sensorineural hearing loss in the left ear. Two weeks later, a repeat MRI showed multiple periventricular hyperintense lesions in the white matter, with corpus callosum involvement, which was highly indicative of SS.

The diagnostic workup of CRAO involves the exclusion of thromboembolic causes. Accordingly, inflammatory and coagulation disorders must also be considered, especially with a unilateral hemi-CRAO. Although emboli and coagulopathy are the most common causes of CRAO, the above cases demonstrate that SS should be included in the differential diagnosis, particularly in younger and healthy patients with this ophthalmic presentation, as the SS clinical triad does not usually present simultaneously^(4)^. Patients with CRAO should undergo a careful examination of the peripheral retina for multiple BRAO.

In conclusion, hemi-CRAO can be considered an atypical neuro-ophthalmological finding related to this disorder. Its presence should increase the suspicion of SS, facilitating its timely diagnosis. Considering the presumed autoimmune etiology of SS, accurate treatment with immunosuppression is necessary. This can improve a patient's visual acuity and eradicate other symptoms^(6)^.
